# U. S. V. M. A.

**Published:** 1895-07

**Authors:** 


### U. S. V. M. A.

INCREASED interest is daily being aroused in the coming meeting of the Association, and the frequent additions to the programme of interesting papers and reports promise to increase very much the attendance. The Pennsylvania State Veterinary Association will probably send as delegates Drs. J. C. Foelker, of Allentown, and John R. Hart, of Philadelphia, both of whom will be found among the party leaving Philadelphia on a special car by the Baltimore and Ohio Railroad. Those contemplating joining the special party destined for Des Moines in September next, and who have not had the opportunity of viewing the beautiful scenery and historic grounds through which the Baltimore and Ohio Railroad runs, will have a great treat in store for them, while those who have enjoyed this beautiful ride will look with anticipated pleasure upon the privilege of again revelling in its beauties.

PRESIDENT HOSKINS has appointed the following members of the Association a local Committee of Arrangements for the meeting at Des Moines in September next: Dr. W. B. Niles, *Chairman*, Ames, Iowa; Dr. M. Stalker, Ames, Iowa; and Dr. F. H. P. Edwards, Iowa City, Iowa.

AMONG those who will attend the meeting at Des Moines, Iowa, are Drs. Huidekoper, Robertson, and Gill, of New York City; Drs. Thomas B. Rayner, Pearson, Hoskins, and Harger, of Philadelphia; Drs. William Dougherty and Clement, of Baltimore.

THOSE who have had the pleasure of listening to the several extremely interesting and instructive papers read by Prof. W. L. Williams at the several meetings of the Association during the past several years will be glad to know that the officers have finally prevailed upon him to favor the Association with a paper at the meeting in Des Moines.

CHAIRMAN TRUMBOWER, of the Committee on Diseases, has addressed, as Chairman of that important Committee, over one hundred of the members of the Association, some sixty State veterinarians and members of the State Live-stock Sanitary Boards, and one hundred members of the State Association of Illinois, asking for information and reports as to the diseases prevalent in their respective localities, and such other information as would be of interest in the proper preparation of his report. Such efforts and labor should be well responded to, not only by those specially addressed, but by every member of the profession, all of whom have had something of interest during the past year.

THE proposition to start a party, by specially chartered car, of veterinarians from the Eastern States for our meeting in September seems to have been uppermost in the minds of several of the strong Association members. As the number is limited to twenty-five, those whose names are first enrolled will be insured a place in this pleasant way of journeying.

THERE will not be a dull hour on the programme at the coming meeting of the Association. Among the latest attractive features will be a paper by Professor Schwarzkopf, "The Horse as a Producer of Antitoxins," a subject of very great importance at this time.

ONE of the features of the coming meeting at Des Moines will be a visit to the veterinary department, at Ames, of the Iowa Agricultural College, at the wishes of the trustees and faculty of the college.

EVERY facility will be afforded Eastern veterinarians to reach Des Moines, aside from the special party which will be recruited along the Baltimore and Ohio Railroad, between New York, Philadelphia, and Baltimore, and which will have a chartered car, making only one change, at Chicago, where a fresh car will be afforded.

Applications for membership are growing more numerous as the time grows closer for our meeting—one of the best signs of progress. They are well distributed geographically, which is always a sign of strength and general interest in Association work.

Among the interesting papers announced for the coming meeting at Des Moines will be one by Prof. S. J. J. Harger, of Philadelphia, on the operation of arytenectomy for roaring. This paper will be directed especially toward determining the value of this operation from a practical point of view and its feasibility as to cost, these points being determined largely by reports obtained from those who have performed the operation.

